# Diabetes Insipidus: Pathogenesis, Diagnosis, and Clinical Management

**DOI:** 10.7759/cureus.13523

**Published:** 2021-02-23

**Authors:** Cody M Mutter, Trevor Smith, Olivia Menze, Mariah Zakharia, Hoang Nguyen

**Affiliations:** 1 Basic Sciences, Nova Southeastern University Dr. Kiran C. Patel College of Osteopathic Medicine, Fort Lauderdale, USA

**Keywords:** central diabetes insipidus, nephrogenic diabetes insipidus, gestational diabetes insipidus, dipsogenic diabetes insipidus, diabetes insipidus, antidiuretic hormone, vasopressin

## Abstract

Diabetes insipidus (DI) is an endocrine condition involving the posterior pituitary peptide hormone, antidiuretic hormone (ADH). ADH exerts its effects on the distal convoluted tubule and collecting duct of the nephron by upregulating aquaporin-2 channels (AQP2) on the cellular apical membrane surface. DI is marked by expelling excessive quantities of highly dilute urine, extreme thirst, and craving for cold water. The two main classifications of DI are central diabetes insipidus (CDI), characterized by a deficiency of the posterior pituitary gland to release ADH, and nephrogenic diabetes insipidus (NDI), characterized by the terminal distal convoluted tubule and collecting duct resistance to ADH. The two less common classifications include dipsogenic DI, characterized by excessive thirst due to a low osmotic threshold, and gestational DI, characterized by increased concentration of placental vasopressinase during pregnancy. Treatment of DI is dependent on the disease classification, but severe complications may arise if not tended to appropriately. The most important step in symptom management is maintaining fluid intake ahead of fluid loss with emphasis placed on preserving the quality of life. The most common treatment of CDI and gestational DI is the administration of synthetic ADH, desmopressin (DDAVP). Nephrogenic treatment, although more challenging, requires discontinuation of medications as well as maintaining a renal-friendly diet to prevent hypernatremia. Treatment of dipsogenic DI is mainly focused on behavioral therapy aimed at regulating water intake and/or administration of antipsychotic pharmaceutical therapy. Central and nephrogenic subtypes of DI share a paradoxical treatment in thiazide diuretics.

## Introduction and background

**Figure 1:**
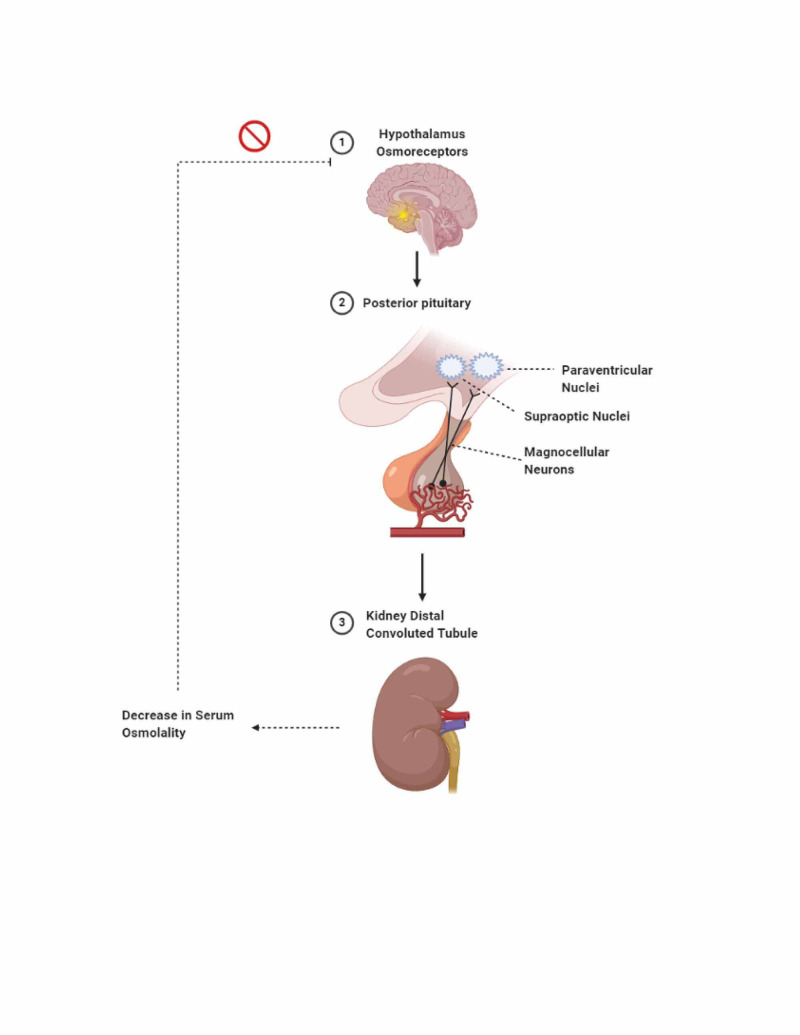
Osmoreceptors in the hypothalamus detect increased serum osmolality. Upon detecting the increased serum osmolality, the hypothalamus sends signals from the supraoptic and paraventricular nuclei to the posterior pituitary via magnocellular neurons to release antidiuretic hormone (ADH) (vasopressin). ADH then reaches the distal convoluted tubules (DCT) of the kidneys and binds to its receptors. This binding causes aquaporin-2 channels to move from the cytoplasm into the apical membrane of the DCT, allowing water to flow back into the bloodstream. As a result, the osmoreceptors in the hypothalamus detect the subsequent decrease in osmolality in the serum and reduces the production of ADH.

**Figure 2:**
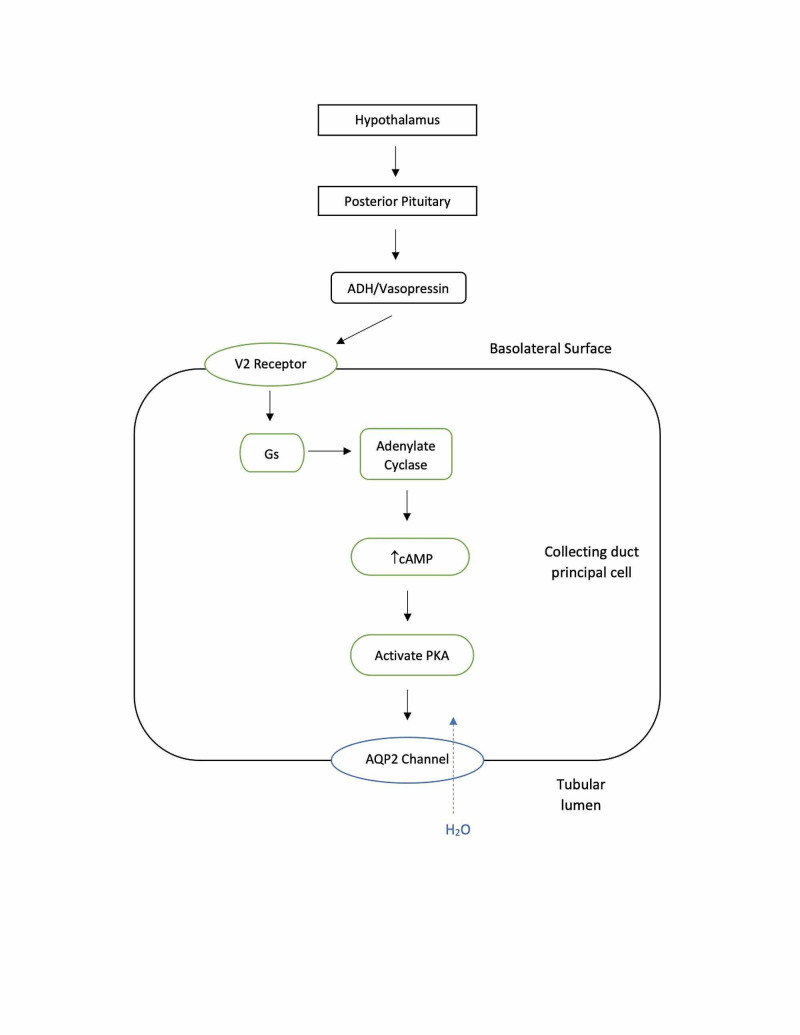
ADH function on cells of the collecting duct. ADH: antidiuretic hormone; AQP2: aquaporin-2 receptors; PKA: protein kinase A.

Diabetes insipidus (DI) is a rare disorder, affecting roughly 1 in 25,000 people or about 0.004% of the global population [1]. Due to the rare occurrence in the population, the various forms of DI can be relatively neglected in medical education as well as in a research setting for improving clinical management [1]. Although DI is an uncommon endocrine disorder the outcome for untreated disease can negatively impact the quality of life for the patient. Epidemiologically, DI does not show a predilection for males or females and it can develop at any age with hereditary forms developing earlier in life [1]. DI can be classified into four major categories which include central, nephrogenic, dipsogenic, or gestational [1]. DI is most commonly defined as a urine volume of more than 3-3.5 liters in a 24-hour period in adults with a urine osmolality of less than 300 mOsmol/kg. In most cases of DI, urine volume far exceeds 3-3.5 liters in a 24-hour period [2]. The principal hormone of diabetes insipidus is the posterior pituitary hormone ADH, which is one of the main determinants regarding water homeostasis within the body. antidiuretic hormone (ADH) acts on its target organ, the kidney, to increase urine osmolality [3]. Osmoregulation and baroregulation are the two principal negative feedback mechanisms that control the secretion of ADH [4]. Ever so slight changes, even that of less than 1% in plasma osmolality, are detected by the osmoreceptors of the hypothalamus. This detection of an increase in osmolality leads to the release of ADH from the posterior pituitary gland. A similar response can be examined with respect to baroreceptors stimulated by a decrease in blood volume. The deviation in blood volume requires approximately a 5%-10% difference in volume [2]. Upon release with its transport protein carrier, neurohypophysin II (NPII) from the hypothalamus, ADH travels to the posterior pituitary where it is stored until released. Once stimulated a change in plasma osmolality or stimulation of baroreceptors, ADH is released into the bloodstream as a water-soluble peptide hormone and acts on its target by binding to the aquaporin-2 receptors (AQP2) in the basolateral membrane of the collecting duct (see Figure 1). Once bound to the receptor, it activates the Gs-adenylyl cyclase system pathway, leading to an increase in intracellular levels of cAMP. This increase in cAMP levels activates protein kinase A, finally leading to the phosphorylation of preformed AQP2 channels. The phosphorylation leads to the insertion of AQP2 into the apical membrane surface of the cell (see Figure 2). It has been established that without this insertion of AQP2 the renal collecting duct would remain essentially impermeable to water. The purpose of AQP2 is to remove water from the renal filtrate and concentrate the urine. In the case of DI, water is unable to move freely from the lumen of the nephron into the cells of the collecting duct along an osmotic gradient, which in turn leads to the excretion of diluted urine. ADH can increase urine osmolality to about 1,200 mOsmol/kg and reduce urine output to 0.5 ml/min or about 700-800 ml/day. Upon establishing water balance within the body, levels of circulating ADH drop and the amount of inserted AQP2 channel proteins in the apical plasma membrane are down-regulated [2,3].

## Review

Etiology

**Figure 3:**
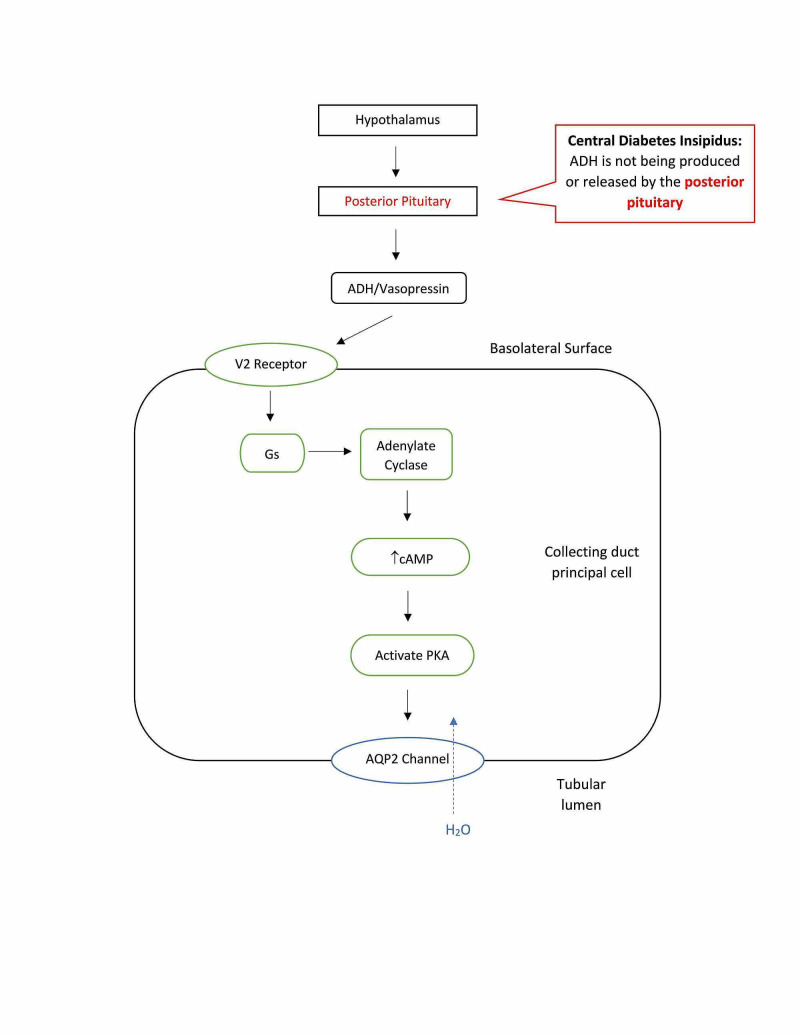
Central diabetes insipidus is due to a deficiency in the production of ADH, often resulting from damage to the pituitary gland. This leads to dilute urine. ADH: antidiuretic hormone; AQP2: aquaporin-2 receptors; PKA: protein kinase A.

The two major forms of DI are central (neurogenic) and nephrogenic. The most common type, central diabetes insipidus (CDI), is due to a deficiency in ADH production (see Figure 3). This is primarily caused by acquired factors such as traumatic brain injuries (TBI), infections, loss of blood to the posterior pituitary or hypothalamus, neurosurgery, and tumors [3]. 25% of CDI cases involve hypothalamo-neurohypophyseal axis lesions [5]. The pituitary gland, the pituitary stalk, and the hypothalamus are quite vulnerable to injury from head trauma, which can result in 16% of CDI cases. 20% of CDI cases are iatrogenic post neurosurgery [5]. Although rare, there are cases of genetic defects in ADH synthesis. These defects can be inherited as autosomal dominant, autosomal recessive, or X-linked recessive traits that can result in CDI. The inherited/familial causes account for 1% of CDI cases [5]. The specific gene mutation most commonly seen is the loss of the AVP gene located on chromosome 20p13 [6]. In addition to the genetic mutation in the AVP gene, there is another rare autosomal recessive disorder that involves DI. This mutation is in the WFS1 gene, which encodes for wolframin. This protein has been shown to function as a transmembrane endoplasmic reticulum element that acts as a calcium channel as well as maintaining the endoplasmic reticulum in pancreatic beta cells [7,8]. The exact mutation in WFS1 leads to Wolfram Syndrome, characterized by AVP-sensitive DI, insulin-dependent juvenile-onset diabetes mellitus, optic atrophy, and sensorineural deafness. DI occurs in ~70% of patients and all four disorders present together in ~50% of patients [9]. Unfortunately, patients presenting with Wolfram Syndrome only survive until the 3rd or 4th decade of life [9]. 

**Figure 4:**
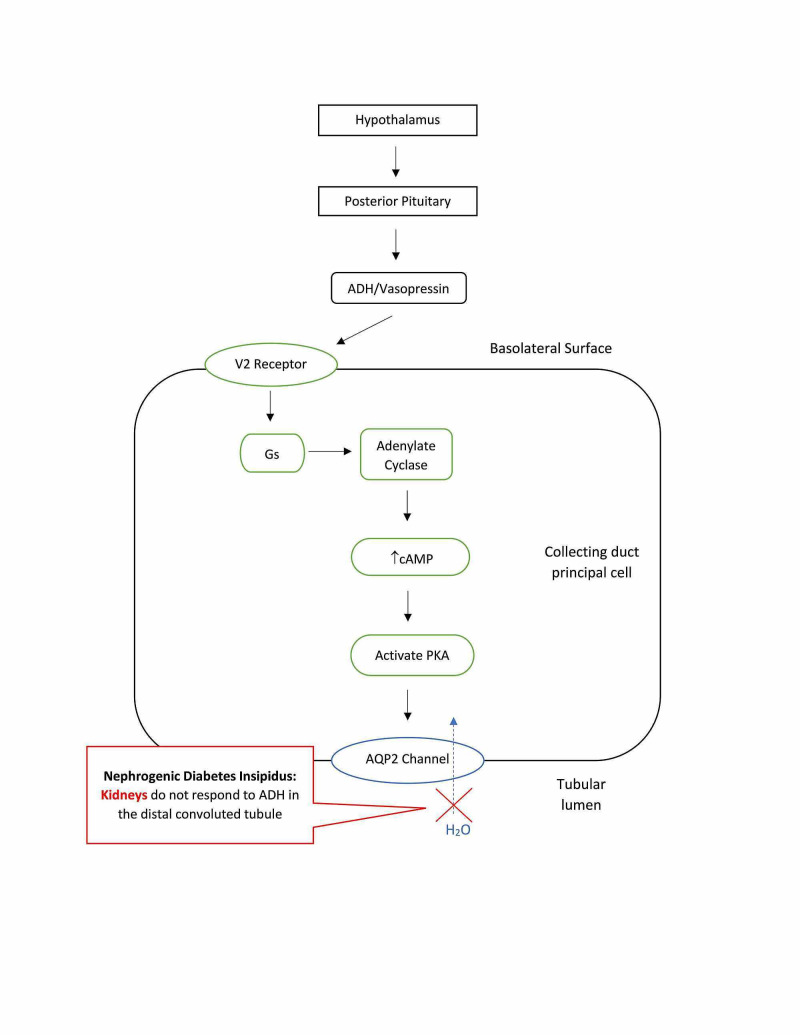
Nephrogenic diabetes insipidus is caused by a defect in the renal tubules. This defect leads to a decreased response to ADH, resulting in dilute urine. ADH: antidiuretic hormone; AQP2: aquaporin-2 receptors; PKA: protein kinase A.

Nephrogenic diabetes insipidus (NDI) is related to the terminal distal convoluted tubule and collecting duct’s insensitivity to circulating ADH (see Figure 4). Most adults with NDI have an acquired abnormality, with the most common causes being lithium therapy or other medications, hypercalcemia, hypokalemia, protein malnutrition, aging, and release of a ureteral obstruction [10]. Lithium therapy is a common practice in treating bipolar disorders. Unfortunately, about 40%-55% of individuals treated with lithium develop the nephrogenic class of DI and can be observed as early as eight weeks after onset of treatment. Lithium is filtered and reabsorbed by the kidney similar to that of sodium and can enter into the collecting duct principal cells. Accumulation of cytotoxic concentrations of lithium within the cells ultimately leads to a decrease in AQP2 expression [10,11]. In addition to lithium therapy resulting in DI, there are reports of other medications causing drug-induced NDI. Foscarnet and clozapine have also been shown to elicit NDI, however, these manifestations are rare and far less common than DI association with lithium [12]. In rare circumstances, the cause of NDI is congenital involving the AQP2 gene. These congenital forms include an X-linked pattern of inheritance (the most common), an autosomal recessive, or an autosomal dominant pattern [10].

**Figure 5:**
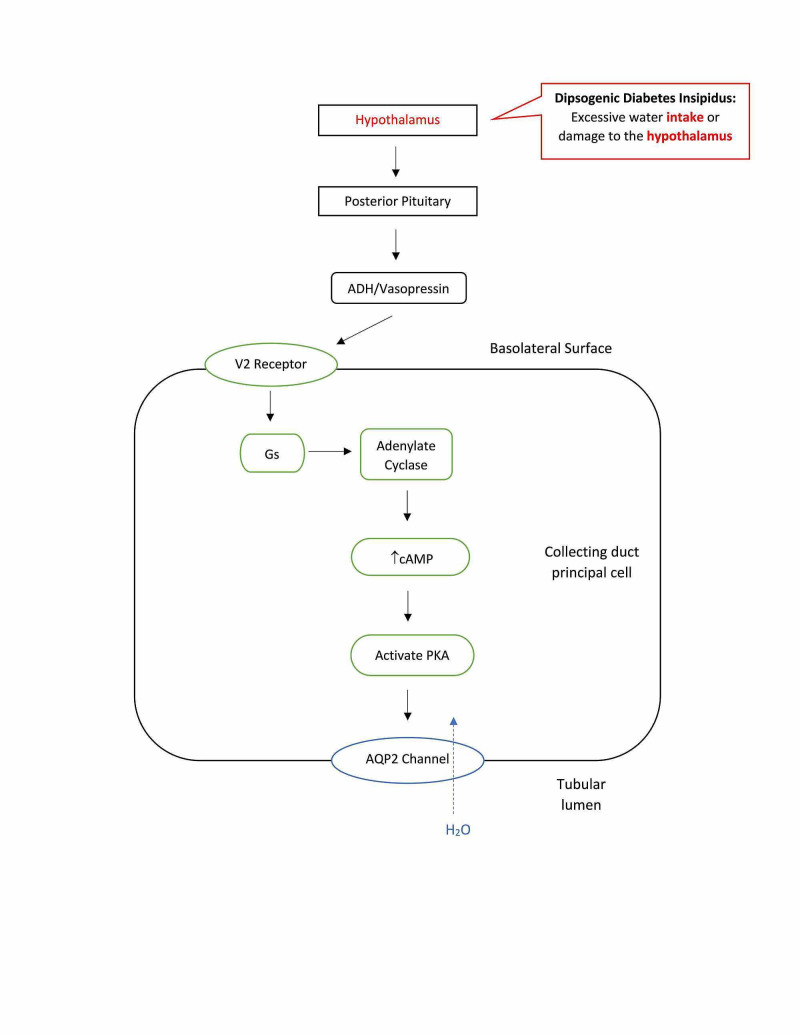
Dipsogenic diabetes insipidus is caused by excessive fluid intake or damage to the thirst-regulating mechanism of the hypothalamus, resulting in dilute urine. ADH: antidiuretic hormone; AQP2: aquaporin-2 receptors; PKA: protein kinase A.

In addition to the two major forms of DI mentioned above, the two less common forms are dipsogenic DI and gestational DI. Dipsogenic DI, also known as primary polydipsia, is classified as having an abnormally low osmotic thirst threshold (see Figure 5) [13]. This leads to increased fluid intake causing physiological suppression of ADH secretion, excretion of large amounts of dilute urine exceeding 40-50 ml/kg body weight, and risk of hyponatremia [14]. In patients with dipsogenic DI, the desire for water decreases after drinking water, but quickly rebounds due to a disrupted oropharyngeal regulation, which is responsible for the physiological suppression of water intake. Unlike nephrogenic and central DI, there is an increase in body water leading to a decrease in plasma osmolarity, but like nephrogenic and central DI there is a decrease in ADH secretion and urine concentration. This form of DI is most commonly seen in patients with psychotic or neurodevelopmental disorders [1]. There are multiple underlying etiologies contributing to the development of dipsogenic DI. These include damage to the hypothalamus, brain injuries, infiltrative or vascular diseases, hippocampus deformations, lesions to certain brain regions such as the amygdala, and stress-reducing behaviors, which release dopamine leading to the secretion of ADH resulting in excessive thirst [14]. Genetics may also play a role in primary polydipsia, where a polymorphism in the orexin 1 receptor has been linked to DI [1].

**Figure 6:**
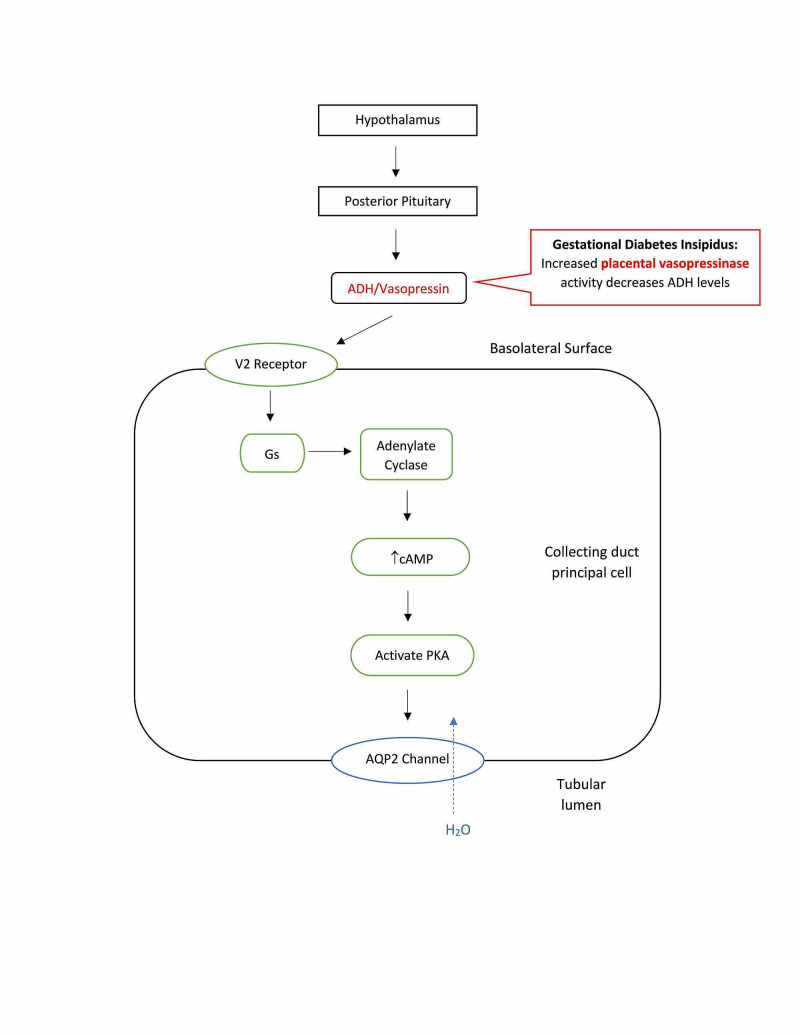
Gestational diabetes Insipidus is caused by an increased concentration of placental vasopressinase, which destroys the mother’s ADH. This leads to large amounts of dilute urine. ADH: antidiuretic hormone; AQP2: aquaporin-2 receptors; PKA: protein kinase A.

Gestational DI occurs due to the rise in placental vasopressinase during pregnancy (see Figure 6). Vasopressinase is an enzyme that degrades ADH resulting in dilute polyuria [15,16]. Placental trophoblasts produce vasopressinase, and the amount produced is proportional to placental size, with twins and multiple pregnancies having the highest levels. Vasopressinase can be detected at 10 weeks and increases approximately 300-fold throughout the pregnancy. Vasopressinase levels are at their highest at the end of the second trimester or beginning of the third, which is when gestational DI most commonly occurs. Women with asymptomatic DI prior to pregnancy may become symptomatic once pregnant because their bodies cannot produce ADH at a rate to replace the ADH being degraded. These patients experience symptoms earlier and with every pregnancy [1,15,16]. During pregnancy, the anterior pituitary becomes enlarged, which compresses the posterior pituitary resulting in decreased release of ADH similar to CDI. The renal tubule also becomes resistant to ADH, as seen in NDI. Progesterone and corticosteroid levels in pregnant women increase causing ADH levels to decrease. Additionally, pregnant women may experience acute fatty liver and HELLP (hemolysis, elevated liver enzymes, and low platelet count) syndrome, which impairs liver function allowing vasopressinase activity to increase because it is not being properly degraded [15,16]. Gestational DI can lead to complications in pregnancy, such as increasing the risk of pre-eclampsia [1]. The subtypes of DI can be compared and summarized in Table 1, including the method of diagnosis and treatment.

**Table 1:** Summary of causes, vasopressin response, diagnosis, and clinical management. DI: diabetes insipidus; ADH: antidiuretic hormone; AVP: arginine vasopressin; AQP2: aquaporin-2 receptors.

	Central DI	Nephrogenic DI	Dipsogenic DI	Gestational DI
Description	Deficiency in release of ADH/AVP from posterior pituitary	Decreased response to ADH/AVP or mutations in AQP2	Abnormally low thirst threshold leading to excessive thirst	Excessive placental vasopressinase
Causes	Brain injury; Infection; Loss of blood to posterior pituitary/hypothalamus; Neurosurgery; Tumor; Genetic defects in ADH synthesis	Lithium therapy; Foscarnet; Clozapine, congenital defect in AQP2 gene; Hypercalcemia; Hypokalemia; Protein malnutrition; Aging	Excessive fluid intake due to psychotic or neuro-developmental disorders; Damage to the hypothalamus; Hippocampus deformations; Brain lesions to the amygdala; Stress-reducing behaviors Genetics	Pregnancy; Genetics; Diet; Sedentary lifestyle
Vasopressin Response	Responds by normalizing urine osmolality	Does not respond/urine osmolality does not change	Does not respond/urine osmolality does not change	Responds by normalizing urine osmolality
Diagnosis	Urine osmolality increases >50% following water deprivation and DDAVP administration; Copeptin <4.9 pmol/L following osmotic stimulation MRI of pituitary gland	Urine osmolality increases <50% following water deprivation and DDAVP administration Baseline copeptin >21.4 pmol/L	Excretion of dilute urine exceeding 40-50 ml/kg of body weight	Serum osmolality greater than 285 mOsm/kg with persistent urine osmolality less than 300 mOsm/L.
Management	DDAVP; Thiazide diuretics; Fluids	Discontinue contributing therapy/medication, Thiazide diuretics, fluids, renal diet (low sodium, protein, and phosphorous)	Behavioral therapy (reduce water intake and balanced diet); Antipsychotic medications	DDAVP

Pathogenesis

Although the initial etiology of each disease is different, all forms lead to the excretion of large volumes of dilute urine, extreme thirst, and severe dehydration. The physiology of water balance in humans is achieved mainly by three interrelated determinants. Those include thirst, ADH synthesis and secretion, and proper kidney function. DI is directly involved with the release of ADH as well as the sensitivity to ADH in the terminal distal convoluted tubule and collecting duct [11]. If the mechanisms of ADH are disrupted, a wide range of changes takes place in the body. Electrolyte imbalances develop, water loss occurs, along with changes in serum and urine osmolality occur. With the onset of the disorder, hypernatremia with serum sodium levels >145 mEq/L (accepted normal range is 135-145 mEq/L) points towards central or nephrogenic DI while a low sodium level points towards primary polydipsia [17,18]. In addition, a serum osmolality >295 mOsm/kg indicates DI while a normal or low serum osmolality (< 285 mOsm/kg) can indicate primary polydipsia [19]. Also, decreased blood volume (hypovolemia), urine osmolality <200 mOsm/kg, decreased urinary sodium level, urinary specific gravity of 1.003 to 1.030, extracellular fluid (ECF) volume, decreased body weight (3%-5%), and initial onset of mild hypertension progressing to hypotension can be observed [20]. Other assessment findings include confusion, irritability, poor skin turgor, and dry mucous membranes [20].

The two principle negative feedback loops associated with body water homeostasis and the effects of DI are quite drastic. The osmoregulation negative feedback loop is in response to changes in serum osmolality, with normal serum osmolality being between 285 mOsm/kg and 295 mOsm/kg. When osmolality is greater than 295 mOsm/kg, a loss of body water has occurred, and the blood is more concentrated. The baroregulation negative feedback loop is in response to changes in blood volume and blood pressure. The hypothalamus responds to the baroreceptor changes by either suppressing or increasing ADH synthesis and release from the posterior pituitary gland. Even slight changes such as a 5-10% decrease in blood volume or a 5% decrease in mean arterial pressure can stimulate ADH release. In general, the body first regulates ADH secretion in response to osmoregulation. In severe volume depletion, baroreceptor stimulation of ADH takes precedence over osmoregulation [21].

Evaluation and differential diagnosis

Clinically, evaluation of a patient involves a thorough history and physical, calculation of plasma osmolality, and total 24-hour urine volume for confirmation of polyuria. Obtaining baseline values of urine osmolality, plasma electrolytes, and random serum are also key during the work-up. The most common presenting signs include polydipsia, polyuria, and nocturia in patients. The differential diagnosis should include hypercalcemia, hypokalemia, sickle cell anemia, histiocytosis, and uncontrolled diabetes mellitus [22].

Diagnosis

**Figure 7:**
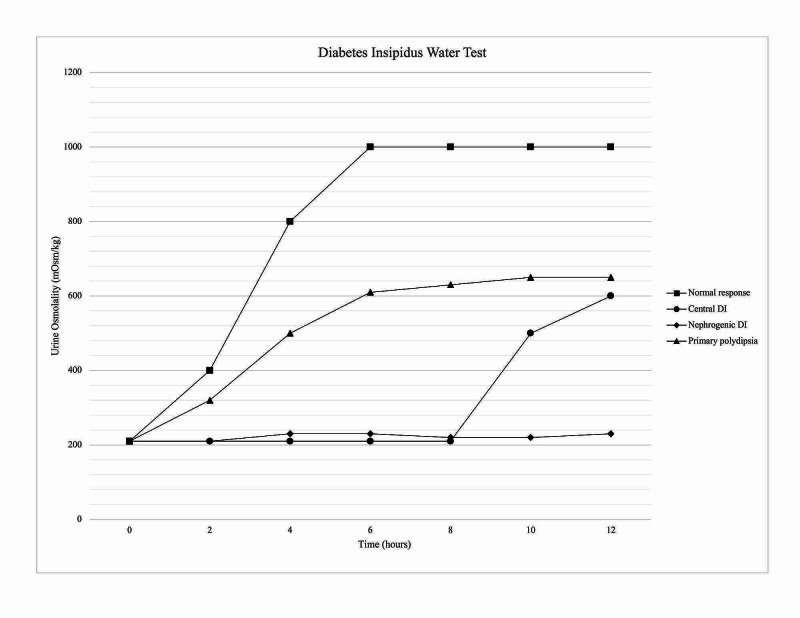
Urine osmolality in water deprivation test. DI: diabetes insipidus.

The indirect water deprivation test involves depriving the patient of fluids and regularly measuring the patient’s urinary excretion, urine osmolality, plasma sodium, and plasma osmolality. The fluid deprivation is continued for either 17 hours maximum, until plasma concentration is greater than or equal to 150 mmol/L, or a loss of 3%-5% of the patient’s body weight has occurred [23]. After exogenous administration of synthetic ADH, or desmopressin (DDAVP), the patient’s urine osmolality is measured to compare to the osmolality before DDAVP administration [24]. At the end of the test, the urine osmolality for healthy individuals should be above 800 mOsm/kg with no increase in urine osmolality following DDAVP. Both nephrogenic and central DI will have urine osmolality below 300 mOsm/kg. The response to DDAVP differentiates nephrogenic and central DI. After DDAVP, urine osmolality will increase >50% for CDI and <50% for NDI [25]. However, the indirect water deprivation test is limited due to its 70% diagnostic accuracy [1]. Although the indirect water deprivation test has been the gold standard for diagnosing dipsogenic DI, the diagnostic accuracy is only 41% [14]. The indirect water deprivation test is not routinely used in pregnancy. If it is used in a pregnant patient, close observation is necessary. Prolonged water restriction could lead to fetal and maternal dehydration, hypernatremia, and increase the risk of uteroplacental insufficiency. Gestational DI is confirmed if serum osmolality is greater than 285 mOsm/kg with persistent urine osmolality less than 300 mOsm/L [16]. Water deprivation test results are graphically represented in Figure 7. 

Zerbe and Robertson proposed a direct AVP measurement to improve accuracy when diagnosing CDI, NDI, or primary polydipsia [26]. Direct AVP measurements involve both depriving the patient of water and osmotically stimulating the patient with hypertonic saline infusion. The AVP levels are then measured and compared to the area of normality. If AVP levels fall above the area, a diagnosis of NDI can be made. If AVP levels fall below the area, a diagnosis of CDI can be made. Primary polydipsia can be diagnosed if the AVP levels fall within normal levels. However, direct AVP measurements only demonstrate 38% accuracy with commercially available assays [27]. Due to test instability and difficult measurements directly measuring AVP levels has not entered clinical practice for diagnosis [14]. To diagnose gestational DI, a vasopressinase inhibitor is given to pregnant women because placental vasopressinase can cause undetectable or falsely elevated AVP levels due to inactive fragment metabolites [16].

Copeptin is the most recent clinical diagnostic marker for DI due to its strong correlation with plasma arginine vasopressin (AVP) [27]. Copeptin and AVP are derived from the same precursor protein, pre-provasopressin [24]. However, copeptin is a better diagnostic marker than AVP because copeptin results can be available in less than two hours with only a small plasma or serum volume of 50 μL required. An increase in systemic osmolality or a decrease in arterial blood volume and pressure triggers the release of both copeptin and AVP [26]. Timper et al. conducted a study to evaluate the diagnostic accuracy of copeptin for various types of DI, which showed that copeptin is a promising new tool in the differential diagnosis of polyuria-polydipsia syndrome as well as an adequate surrogate marker for AVP [17]. Prior to osmotic stimulation, the patients with a baseline copeptin of >21.4 pmol/L had NDI. If baseline copeptin is below 21.4 pmol/l, osmotic stimulation is needed to distinguish between CDI and primary polydipsia. Following osmotic stimulation (water deprivation and 3% saline infusion), patients with primary polydipsia had a copeptin level >4.9 pmol/L, and patients with CDI had a copeptin level <4.9 pmol/L. The diagnostic accuracy was 96% [17]. A follow-up study with 156 patients with polyuria polydipsia syndrome was conducted [28]. The patients were osmotically stimulated with only a hypertonic saline infusion. Once the patients’ serum sodium was at least 150 mmol/L, the copeptin levels were measured and showed 97% accuracy [28]. More research is needed to create a set range for copeptin levels to indicate gestational DI. Increased levels of copeptin in the third trimester can increase the risk of complications during pregnancy, such as preeclampsia [16]. An MRI of the pituitary gland can also be used to diagnose CDI. The MRI for a patient with CDI will classically have thickening of the infundibular stalk and lack normal T1 posterior pituitary bright spot (PPBS). However, early stages of CDI may present with PPBS [5].

Treatment

Treatment of DI is crucial to improving the quality of life of the patient. The initial cause of the disorder determines whether or not symptoms can be fully alleviated or treated. Both central and nephrogenic DI have a few first-line treatments that help maintain fluid balance. Always having access to water is of utmost importance to prevent being overly dehydrated too quickly. A paradoxical treatment that is used to manage CDI and NDI is the use of thiazide diuretics, which inhibit the NaCl cotransporter in the renal distal convoluted tubule [29-32]. This portion of the nephron is impermeable to water and considered to be part of the diluting segment. Therefore, the water preserving effect of thiazide diuretics is unlikely related to a direct effect on the distal convoluted tubule [32]. The most widely accepted hypothesis suggests that the antidiuretic action is secondary to increased renal sodium excretion. The renal sodium loss causes extracellular volume contraction leading to lowered glomerular filtration rate (GFR) and increased proximal tubular sodium and water reabsorption. Other treatment approaches differ by principal type. 

The treatment of choice for CDI is the administration of synthetic ADH, also known as desmopressin or DDAVP. DDAVP is a synthetic analog of the endogenous hormone ADH, but with a 2,000-3,000 fold lower antidiuretic effect. DDAVP can be administered orally, intranasally, or parenterally. The most effective route seems to be intranasal or oral, as plasma concentrations are reached within 40-55 minutes. Generally, urine output will decrease one to two hours after administration and the duration of action will range from 6 to 18 hours. Rare side effects with intranasal delivery of DDAVP include eye irritation, headache, dizziness, rhinitis or epistaxis, coughing, flushing, nausea, vomiting, abdominal pain, chest pain, palpitations, and tachycardia [11,29].

Beyond water consumption and the administration of thiazide diuretics that help alleviate symptoms, NDI is slightly more complicated. The majority of acquired cases of this rare disease develop from patients being treated with lithium for bipolar disorder [10,30]. The complications with lithium therapy arise with prolonged use. Prolonged lithium therapy can lead to irreversible nephrogenic diabetes insipidus even after lithium therapy has been withdrawn. The use of DDAVP is not effective because the initial disorder lies in the nephron and its insensitivity to ADH, not a deficiency in the release of ADH [10,30]. However, new advances in treatment for nephrogenic diabetes insipidus are currently underway. Studies involving mice have shown that secretin increased AQP2 levels in cells [33]. The addition of Fluvastatin led to the AQP2 being taken to the plasma membrane, possibly indicating that this combination could be used as a pharmacologic target for treating NDI [33]. As a result, this sparked new research into the role of statins in treating NDI. A recent double-blind, randomized, placebo-controlled, pilot trial evaluating the efficacy of atorvastatin in NDI patients using lithium did not show significant improvement in urine osmolality over 12 weeks [34]. Further trials with longer follow-up may help assess the effectiveness of atorvastatin on NDI. Further investigation on the biological mechanisms of atorvastatin in controlling symptoms of NDI may allow psychiatric patients to safely utilize lithium therapy [34]. Other helpful treatments involve proper dietary practices in which a sodium restriction or renal diet is put into action via the dietician or physician [10,30].

The ideal approach for managing dipsogenic DI is behavioral therapy to decrease voluntary water intake, however, this is difficult because the patient suffers from excessive thirst. The patient can be educated on the disease, attend group therapy, and use biofeedback for relaxation training. There are also support measures that can be established such as a balanced diet, avoiding dry mouth causing drugs, and checking weight to see if water is being retained [14]. Additionally, antipsychotic drugs can be used to prevent hyponatremia and improve polydipsia behavior. These drugs include lithium, olanzapine, clozapine, risperidone, phenytoin, bupropion, and propranolol. Dipsogenic DI can cause hyponatremia, which is treated with water restriction, but in serious cases can be treated with a 3% saline infusion [14].

DDAVP is the treatment of choice because it is resistant to placental vasopressinase, and other methods are not as successful due to increased vasopressinase levels. This resistance comes from an altered arginine located at the 8th position [16]. In addition, DDAVP is more selective for the AVPR2 activator resulting in less oxytocic activity, which decreases stimulation causing uterine contractions [16]. Intranasally is the preferred method of administration. DDAVP in pregnancy is classified as a class B teratogen, having minimal side effects on both the mother and fetus. Later in pregnancy, a higher dose may be needed due to increased placental vasopressinase levels. Following pregnancy, DDAVP can still be given at lower doses or completely stopped. DDAVP does not affect lactation because it does not enter breast milk [16]. Previous work has shown that DDAVP use is safe and efficacious, with no adverse neonatal effects [35]. Hypernatremia must also be corrected in a critical care setting with observation and controlled fluid resuscitation of 1mmol/L/h. However, oligohydramnios has been reported as a rare complication [36,37]. Table 1 provides a summary of the principal types of DI, causes, vasopressin response, diagnosis, and clinical management. 

Prognosis and prevention

Depending on the underlying cause of DI, the quality of life following disease onset and treatment can be highly variable. Although the identification of the principal type and causes of DI have become more recognizable, the great variety and severity of the disorder and the genetic basis means that no current treatment regime exists that fully alleviates symptoms in all patients. In cases where CDI is brought on by severe trauma or head injury, DI not only leads to lower quality of life, but the initial trigger for the disorder may create a plethora of other complications for the patient and the family. For patients in which the cause is malignancy, the prognosis is guarded relative to benign causes [22]. NDI can be fully alleviated if damage to the nephron is not extensive, which can be observed in prolonged lithium therapy. If the medication is discontinued early in disease onset the extent of nephron damage may be much less and easily handled. DDI can also be fully cured if damage to the hypothalamus or pituitary is not extensive. Damage can be caused by surgery, inflammation, infection, head injury, or a tumor. It can also be cured if the underlying mental illness causing the excessive thirst is properly treated [14]. 

Lastly, gestational DI can only occur during pregnancy when the placenta produces vasopressinase. Most women will not need treatment following delivery; however, they may develop gestational DI with additional pregnancies and are at a higher risk of developing type 2 diabetes mellitus [38]. As stated previously, if damage is extensive there is no cure for permanent damage that creates an irreversible form. Depending on the severity of the disorder, making sure adequate fluid intake meets excretion, maintaining adequate therapy through DDAVP, adhering to a renal diet, monitoring weight loss, and therapy through thiazide diuretics can create a fairly tolerable life. Poorly managed DI can be life-threatening. 

There is no specific pattern, gender, or race that is immediately more susceptible to acquired DI. As can be seen with CDI and DDI, the acquired forms develop from traumatic brain injury, infections, surgery, cancer, and hypothalamus/posterior pituitary hemorrhage. DDI can also be caused by mental illness such as schizophrenia [14]. The main form of NDI is acquired through lithium therapy in bipolar patients [2, 10]. Other medications leading to NDI include amphotericin B and demeclocycline, but are extremely rare [2,10]. In congenital CDI, the frequency of autosomal forms is currently unknown. Regarding congenital NDI, inheritance follows an x-linked pattern. In particular, x-linked NDI accounts for approximately 90% of congenital NDI and occurs with a frequency of 4-8 per one million live male births. No gender difference has been reported for the autosomal dominant and recessive forms [2,10,11]. Interestingly, women who are pregnant with males are at a higher risk of developing gestational DI [39]. However, the main contributing factors leading to gestational DI are genetics combined with sedentary lifestyle factors. 

## Conclusions

Albeit uncommon, the consequences of untreated DI can place a significant burden on the patient and negatively impact the quality of life. Appropriate diagnosis and therapeutic intervention are critical to ensure improved quality of life for the patient. A deficiency in the release of ADH from the posterior pituitary gland leads to CDI. In NDI, the tubular cells of the collecting duct no longer respond to the action of ADH. Similar to CDI, dipsogenic DI and GDI can be characterized by a deficiency in ADH. Dipsogenic DI is due to an abnormally low osmotic thirst threshold, leading to increased fluid intake. GDI is characterized by a rise in placental vasopressinase, leading to the degradation of ADH in the mother. Diagnosing the various types of DI relies primarily on measuring urine osmolality following water deprivation, vasopressin response, and copeptin measurement following osmotic stimulation. Diagnosis of CDI and NDI can be made with urine osmolality following water deprivation and DDAVP administration, and copeptin. MRI of the brain can also be useful in the workup for CDI. Dipsogenic DI diagnosis can be made when there is an excretion of dilute urine with an abnormal osmotic thirst threshold. Diagnosing GDI can be made by measuring serum and urine osmolarity. Management of DI is rooted in improving patient quality of life and counteracting extreme fluid floss. Treating CDI includes the administration of DDAVP along with adequate fluid intake. Paradoxically, both CDI and NDI can be managed by thiazide diuretics. Primary management of NDI includes discontinuing the offending agent, such as Lithium. It should be noted that NDI, along with dipsogenic DI, does not respond to DDAVP administration. Primary management of dipsogenic DI is focused on behavioral therapy to reduce water intake, as well as the inclusion of an antipsychotic medication if warranted. DDAVP is the principal treatment option for GDI. Prognosis of each type is usually excellent, as adequate treatment leads to markedly improved quality of life for patients.

## References

[1] Christ-Crain M, Bichet DG, Fenske WK, Goldman MB, Rittig S, Verbalis JG, Verkman AS (2019). Diabetes insipidus. Nat Rev Primers.

[2] Moeller HB, Rittig S, Fenton RA (2013). Nephrogenic diabetes insipidus: essential insights into the molecular background and potential therapies for treatment. Endocrine Rev.

[3] Robertson GL (2001). Antidiuretic hormone: normal and disordered function. Endocrinology.

[4] Hickey J Fluid and metabolic disorders in neuroscience patients. The Clinical Practice of Neurological and Neurosurgical Nursing.

[5] Adams NC, Farrell TP, O’Shea A (2018). Neuroimaging of central diabetes insipidus—when, how and findings. Neuroradiology.

[6] Schernthaner-Reiter MH, Stratakis CA, Luger A (2017). Genetics of diabetes insipidus. Endocrinol Metabol Clin N Am.

[7] Osman AA, Saito M, Makepeace C, Permutt MA, Schlesinger P, Mueckler M (2003). Wolframin expression induces novel ion channel activity in endoplasmic reticulum membranes and increases intracellular calcium. J Biol Chem.

[8] Fonseca SG, Fukuma M, Lipson KL, Nguyen LX, Allen JR, Oka Y, Urano F (2005). WFS1 Is a novel component of the unfolded protein response and maintains homeostasis of the endoplasmic reticulum in pancreatic β-cells. J Biol Chem.

[9] Barrett TG, Bundey SE, Macleod AF (1995). Neurodegeneration and diabetes: UK nationwide study of Wolfram (DIDMOAD) syndrome. Lancet.

[10] Sands JM, Bichet DG (2006). Nephrogenic diabetes insipidus. Ann Intern Med.

[11] Iorgi ND, Napoli F, Allegri AEM (2012). Diabetes insipidus—diagnosis and management. Hormone Res Paediatrics.

[12] Bendz H, Aurell M (1999). Drug-induced diabetes insipidus: incidence, prevention and management. Drug Safety.

[13] Perkins RM, Yuan CM, Welch PG (2006). Dipsogenic diabetes insipidus: report of a novel treatment strategy and literature review. Clin Exp Nephrol.

[14] Sailer CO, Winzeler B, Christ-Crain M (2017). Primary polydipsia in the medical and psychiatric patient: characteristics, complications and therapy. Swiss Medical Weekly.

[15] Ananthakrishnan S (2016). Diabetes insipidus during pregnancy. Best Pract Res Clin Endocrinol Metab.

[16] Ananthakrishnan S. (2020). Gestational diabetes insipidus: diagnosis and management. Best Pract Res Clin Endocrinol Metab.

[17] Timper K, Fenske W, Kühn F (2015). Diagnostic accuracy of copeptin in the differential diagnosis of the polyuria-polydipsia syndrome: a prospective multicenter study. J Clin Endocrinol Metab.

[18] Christ-Crain M, Fenske W (2016). Copeptin in the diagnosis of vasopressin-dependent disorders of fluid homeostasis. Nat Rev Endocrinol.

[19] Fenske W, Quinkler M, Lorenz D (2011). Copeptin in the differential diagnosis of the polydipsia-polyuria syndrome—revisiting the direct and indirect water deprivation tests. J Clin Endocrinol Metab.

[20] Simerville JA, Maxted WC, Pahira JJ (2005). Urinalysis: a comprehensive review. Am Fam Physician.

[21] John CA., Day MW (2012). Central neurogenic diabetes insipidus, syndrome of inappropriate secretion of antidiuretic hormone, and cerebral salt-wasting syndrome in traumatic brain injury. Critical Care Nurse.

[22] Hui C, Radbel JM (2020). Diabetes insipidus. StatPearls.

[23] Refardt J, Winzeler B, Christ-Crain M (2020). Diabetes insipidus. Endocrinol Metab Clin N Am.

[24] Refardt J, Winzeler B, Christ‐Crain M (2019). Copeptin and its role in the diagnosis of diabetes insipidus and the syndrome of inappropriate antidiuresis. Clin Endocrinol.

[25] Refardt J (2020). Diagnosis and differential diagnosis of diabetes insipidus: update. Best Pract Res Clin Endocrinol Metab.

[26] Zerbe RL, Robertson GL (1981). A comparison of plasma vasopressin measurements with a standard indirect test in the differential diagnosis of polyuria. N Engl J Med.

[27] Christ-Crain M (2020). Diabetes insipidus: new concepts for diagnosis. Neuroendocrinology.

[28] Fenske W, Refardt J, Chifu I (2018). A copeptin-based approach in the diagnosis of diabetes insipidus. N Engl J Med.

[29] Bichet D, Sterns RH, Emmett M, Wolfsdorf JI (2019). Treatment of Central Diabetes Insipidus.

[30] Bichet D., Sterns R. H., Mattoo T. K. (2019). Treatment of Nephrogenic Diabetes Insipidus. UpToDate, Forman, J. P..

[31] (2020). Diabetes Insipidus | NIDDK. (n.d.). https://www.niddk.nih.gov/health-information/kidney-disease/diabetes-insipidus.

[32] Kim GH (2004). Antidiuretic effect of hydrochlorothiazide in lithium-induced nephrogenic diabetes insipidus is associated with upregulation of aquaporin-2, Na-Cl Co-transporter, and epithelial sodium channel. J Am Soc Nephrol.

[33] Procino G, Milano S, Carmosino M (2014). Combination of secretin and fluvastatin ameliorates the polyuria associated with X-linked nephrogenic diabetes insipidus in mice. Kidney Int.

[34] Fotso Soh J, Beaulieu S, Trepiccione F (2020). A double-blind, randomized, placebo-controlled pilot trial of atorvastatin for nephrogenic diabetes insipidus in lithium users. Bipolar Disorders.

[35] Ray JG (1998). DDAVP use during pregnancy: an analysis of its safety for mother and child. Obstet Gynecol Surv.

[36] Hanson RS, Powrie RO, Larson L (1997). Diabetes insipidus in pregnancy: a treatable cause of oligohydramnios. Obstet Gynecol.

[37] Choi HS, Kim YH, Kim CS, Ma SK, Kim SW, & Bae EH (2018). Diabetes insipidus presenting with oligohydramnios and polyuria during pregnancy. J Nippon Med School.

[38] Noctor E (2015). Type 2 diabetes after gestational diabetes: the influence of changing diagnostic criteria. World J Diab.

[39] Quigley J, Shelton C, Issa B, Sripada S (2018). Diabetes insipidus in pregnancy. Obstet Gynaecol.

